# Principles of thesis writing

**Published:** 2008-06

**Authors:** Rita Sood

**Affiliations:** Department of Medicine, All India Institute of Medical Sciences, Ansari Nagar, New Delhi -110029, India. E-mail: ritasood@gmail.com

This is the first edition of this book, authored by the three editors, all of whom are Pediatricians. It has 93 pages and 11 chapters besides a glossary, suggested readings and an index. The authors have assumed the task of providing a book that attempts to cover the process of thesis writing in a simplified and step wise approach.

The first chapter is introductory and deals with the need for research, scientific approach in thinking, training in research methodology and thesis writing during the Postgraduate Training in Medicine.

The next few chapters deal with the basics of research methodology including formulating a research problem, the types of study design and the sampling techniques. There is a comprehensive though crisp introduction to various search engines for an effective and focused search in literature. There is a chapter devoted to the writing and presentation of thesis protocol, guiding the postgraduates into the finer details of writing and presentation. Tips have also been given for an effective presentation of research protocol in a limited period of time. Though at places, formulae could be intimidating, an effort has been made to present the basics of statistics and presentation of data in a simplified way, clarifying to the reader concepts behind these formulae.

The postgraduates will find the last three chapters on graphical presentation of data, writing of references and the process of submission of thesis most useful when they start writing their work in the form of a thesis. In the end, there is a comprehensive glossary of terms, suggested readings and an index simplifying the use of this book.

This book covers almost all aspects of thesis writing a student is likely to encounter from the time of deciding the topic to the final submission of thesis. The most complementary feature of this book is the simplicity of language, at places conversational, making it very user friendly. There is an abundant use of examples making it still simpler to follow. 

The book is useful not only for the postgraduates but also for the young faculty who are beginning to guide postgraduates in thesis writing. Though not significant, there are few spelling mistakes which can be easily overlooked. The book is a “must have”for every postgraduate student in health professional education.

